# Diagnostic utility of GDF15 in neurodegenerative diseases: A systematic review and meta‐analysis

**DOI:** 10.1002/brb3.2502

**Published:** 2022-01-24

**Authors:** Xin‐Hong Xue, Lin‐Lin Tao, Dao‐Qing Su, Cun‐Ju Guo, Hong Liu

**Affiliations:** ^1^ Department of Neurology Liaocheng Hospital Affiliated to Shandong First Medical University Liaocheng People's Hospital Liaocheng China; ^2^ Department of Neurosurgery Liaocheng Hospital Affiliated to Shandong First Medical University Liaocheng People's Hospital Liaocheng China

**Keywords:** diagnostic utility, GDF15, meta‐analysis, neurodegenerative diseases

## Abstract

**Introduction:**

GDF15 may be a potential biomarker for neurodegenerative diseases. In this analysis, we aimed to quantitative analysis the levels of GDF15 in patients with neurological diseases and in health control, and then to determine its potential diagnostic utility.

**Methods:**

Two researchers separately conducted a systematic search of the relevant studies up to January 2021 in Embase, PubMed, and Web of Science. Effect sizes were estimated to use the standardized mean difference (SMD) with 95% confidence interval (CI). Sensitivity and specificity were calculated by the summary receiver operating characteristics curve (SROC) method. The sensitivity analysis was performed by the “one‐in/one‐out” approach. Considering the considerable heterogeneity among studies, random‐effects model was used for the meta‐analysis investigation.

**Results:**

A total of eight articles were included in this meta‐analysis and systematic review. The pooled results of the random effect model indicated GDF15 levels were significantly higher in patients with neurodegenerative disease than healthy people (SMD = 0.92, 95% CI: 0.44–1.40, *Z *= 3.75, *p *< 0.001). Sensitivity and specificity of biomarker of GDF15 were 0.90 (95% CI: 0.75–0.97), 0.77 (95% CI: 0.67–0.65), and AUC = 0.87 (95% CI: 0.84–0.90), respectively.

**Conclusions:**

GDF15 levels were higher in patients with neurodegenerative disease than healthy people. And serum levels of GDF15 were a better marker for diagnostic utility of neurodegenerative disease.

## Graphical abstract

Our results found that GDF15 levels were higher in patient with neurodegenerative disease than that in the healthy people. And serum levels of GDF15 were a better marker for diagnostic utility of neurodegenerative diseases.

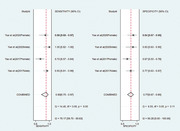

## 1 INTRODUCTION

Neurodegenerative diseases have become a major threat to human health. The prevalence of age‐related disorders was increasing with the aging population worldwide in recent years (Heemels, 2016). Neurodegenerative diseases included mild cognitive impairment (MCI), Alzheimer's disease (AD), Parkinson's disease, multiple system atrophy (MSA), etc. These diseases are numerous in their pathophysiology, with some causing memory and cognitive decline, while others affect a person's ability to move, speak, and breathe (Abeliovich & Gitler, 2016; Canter et al., 2016; Fanciulli et al., 2019). These diseases were characterized by neurodegenerative changes and there was currently no effective cause‐related treatment. Thus, biomarkers are urgently needed for early diagnosis, prognostic prediction of disease progression, and monitoring of the treatment response.

Growth differentiation factor 15 (GDF15) is a branch of the transforming growth factor‐β (TGF‐β) superfamily. It is also known as macrophage inhibitory cytokine‐1(MIC‐1) (Strelau et al., 2000). These family members play an essential role in development, cell multiplication, differentiation, and repair. GDF15 was widely existed in the central nervous system and peripheral nervous system, most prominent in the choroid plexus, and secreted into the cerebrospinal fluid (Strelau et al., 2000). GDF15 is mainly generated in the choroid plexus, damaged neurons, and microglial cells in the central nervous system (Unsicker et al., 2013). In the peripheral nervous system, the main source of GDF15 is Schwann cells (Strelau et al., 2009). Because the concentration of GDF15 protein in the circulation can be easily measured, serious studies have explored it as a significant plasma biomarker associated with various diseases. GDF15 is correlated with cardiovascular diseases such as atrial fibrillation, heart failure, and coronary artery disease (Emmerson et al., 2018). GDF15 levels in patients with cancer, diabetes, cognitive impairment, and cachexia were also identified increasing. Hagström et al. (2017) assessed the levels of GDF15 in 14,577 patients with stable coronary heart disease and showed that the levels of GDF15 to be correlated with heart failure death, cardiovascular death, cancer death, sudden death, and hospitalization for heart failure. Brown et al. (2003) determined serum GDF15 levels from 193 patients with colorectal carcinoma or adenomatous polyps and 260 healthy blood donors. They found that GDF15 levels increased in patients with adenomatous polyps or colorectal cancer (Brown et al., 2003).

In additions, abnormal concentrations of GDF15 are also believed to be closely correlated with neurodegenerative diseases and may be a potential biomarker for early diagnosis and prognosis (Kim et al., 2015). According to animal model studies, GDF15 might play a positive role in stress response and contribute to neurogenesis (Carrillo‐García et al., 2014; Kim et al., 2015). Yao et al. (2017) concluded that GDF15 levels of the patients with PD were significantly higher than those of the healthy controls. Meanwhile, increased GDF15 levels were reported in patients with MSA, AD, and cognitive impairment (Chai et al., 2016; Yue et al., 2020). However, a study by Conte et al. (2020) suggested GDF15 levels were similar between AD patients and healthy control.

Therefore, we aimed to quantitative analysis the levels of GDF15 in patients with neurological diseases and in health control, and then to determine its potential diagnostic utility.

## 2 METHODS

This meta‐analysis was performed following the preferred reporting items for systematic reviews and meta‐analysis (PRISMAs) statement (Moher et al., 2015). All analyses were based on previously published studies; thus, ethical approval or patient consent was not suitable for this meta‐analysis.

### 2.1 Search strategy and studies selection

Two researchers (Xin‐hong Xue and Lin‐Lin Tao) separately conducted a systematic search of the relevant studies up to January 2021 in Embase, PubMed, and Web of Science. Keywords utilized were “growth differentiation factor 15″ OR “GDF‐15″ OR “Macrophage Inhibitory Cytokine‐1″ OR “MIC‐1″ OR “MIC1,” in combination with (“neurodegenerative disease”) OR (“Parkinson's disease” OR “PD”) OR (“MSA” OR “multiple system atrophy”) OR (“Dementia with Lewy body” OR “DLB”) OR (“Alzheimer disease” OR “ Dementia” OR “AD” OR “MCI” OR “mild cognitive impairment”) OR (“Progressive paralysis” OR “PSP”). These terms can be found everywhere in the text of the manuscript. Further literature was searched by scanning the reference list of retrieved studies. To reduce publication bias, peer‐reviewed papers and unpublished (e.g., abstract of posters or oral presentations) studies were considered. Only English studies were considered appropriate.

### 2.2 Eligibility criteria

Two researchers (Xin‐hong Xue and Lin‐Lin Tao) independently assessed the title and abstract of the eligible studies, and disagreements were discussed with the third investigator to reach a consensus. Studies were included in the analysis if they satisfied the following criteria: (1) the study was a diagnostic study investigating GDF15 in patients with neurodegenerative disease, containing AD, MAS, PSP, etc.; (2) the control group of study was selected as the healthy control group; (3) the study reported the serum level of GDF15 and diagnostic validity of neurodegenerative disease and health. We also included studies without sufficient data in a systematic review but excluded them from the summary of the key point.

The following exclusion criteria were used: (1) the study reported a single case study; (2) duplication studies; (3) study subjects were animals; and (4) the study was systematic review and meta‐analysis.

### 2.3 Data extraction and study quality

Two researchers independently scanned the full text of included studies and then extracted data. The extracted data were compared to make certain that information was correctly assessed. The following information were extracted: author's name, published the year of study, the number of patients, age of patients, serum level of GDF15, diagnostic validity, type of disease (e.g., PD, MSA, AD, MCI, etc.). The quality of the included studies for this analysis was evaluated utilizing the Newcastle–Ottawa Scale (NOS) (Stang, 2010). This scale contains three section: (1) selection (3 items, maximum score: 3 points); (2) comparability (1 item, maximum score: 2 points); (3) outcome (2 items, maximum score: 3 points). The studies were divided into one of the following categories: very good studies: 7–8 points; good studies: 5–6 points; satisfactory studies: 3–2 points; unsatisfactory studies: 0–1 points.

### 2.4 Statistical analysis

Analyses were performed using the STATA15.0 software. Effect sizes were estimated using the standardized mean difference (SMD) with 95% confidence interval (CI). *I* ^2^ statistical analysis and *Q* test were calculated to explore heterogeneity between studies. Sensitivity and specificity were calculated by the summary receiver operating characteristics curve (SROC) method. Besides, a summary estimate of the sensitivity and specificity, which includes 95% CI, was provided. Less than 10 studies were included; a funnel plot was not used to explore publication bias. The sensitivity analysis was performed by the “one‐in/one‐out” approach. Considering the considerable heterogeneity between studies, a random‐effects model was used for the meta‐analysis investigation.

## 3 RESULTS

### 3.1 Baseline characteristics of studies

**FIGURE 1:**
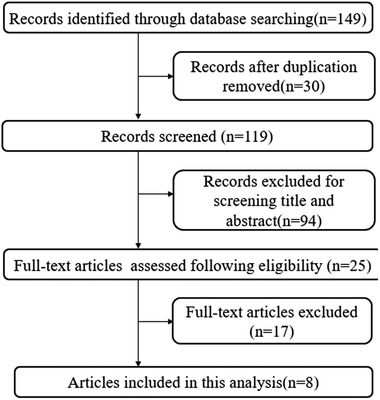
Flow chart for the systematic selection of articles

Overall, we searched 149 articles, 30 articles were removed because of duplication, and 94 articles were removed on the basis of their title and abstract. A total of 25 full‐text articles were assessed; 8 articles were included based on the criteria. The detailed retrieval process was shown in Figure 1. The baseline characteristics of studies were shown in Table 1. The eligible studies were published between 2013 and 2020. The ages of all participants included in the study were over 50 years old. The disease type of included studies contained PD, MSA, AD, and cognitive impairment. The quality of studies was assessed by NOS and the results were presented in Table 1. With NOS quality criteria, all the studies scored 6 or above and had good qualities.

**TABLE 1:** Baseline characteristic of included studies and quality of studies

Authors (year)	Disease	Design	Sampling	Age	GDF15 (pg/ml)	ROC	Sensitivity	Specificity	Quality
Maetzler et al. (2016)	Parkinson's disease dementia	Cross‐sectional	17	75 (61–84)	200 (67–467)	–	–	–	7
	Health control		95	61 (38–79)	184 (39–461)	–	–	–	
Miyaue et al. (2020)	Parkinson's disease	Case‐control	36	72.44 ± 8.44	1472.22 ± 820.12	–	–	–	8
	Health control		30	71.39 ± 8.86	1092.83 ± 543.97	–	–	–	
Davis et al. (2020)	Parkinson's disease	Case‐control	121	63.6 ± 9.9	2603.1 ± 192.7	.57	–	–	8
	Health control		103	61.4 ± 12	2542.7 ± 205.8	–	–	–	
Yao et al. (2017)	Parkinson's disease	Case‐control	104	59.22 ± 8.14	573.50 ± 246.64	.86	71.15%	87.50%	7
	Health control		88	58.33 ± 8.31	288.30±133.32	–	–	–	
Yue et al. (2020)	MSA	Case‐control	49	61.7 ± 7.7	1105.69 ± 984.24	.929	87.50%	88.00%	7
	Health control		50	61.4 ± 7.5	313.85 ± 247.76	–	–	–	
Conte et al. (2020)	Alzheimer's disease	Case‐control	120	52–87	–	–			7
	Health control		92	60–87	–	–	–	–	
Fuchs et al. (2013)	Cognitive impairment	Cohort study	888	78.5 ± 4.7	–	–	–	–	6
Chai et al. (2016)	Cognitive impairment and dementia	Case‐control	100	77.4 ± 7.1	1606.4 (1486.5)	–	–	–	8
	Health control		80	68.3 ± 5.9	827.1 (403.0)	–	–	–	

### 3.2 Association between GDF15 and neurodegenerative disease

**FIGURE 2:**
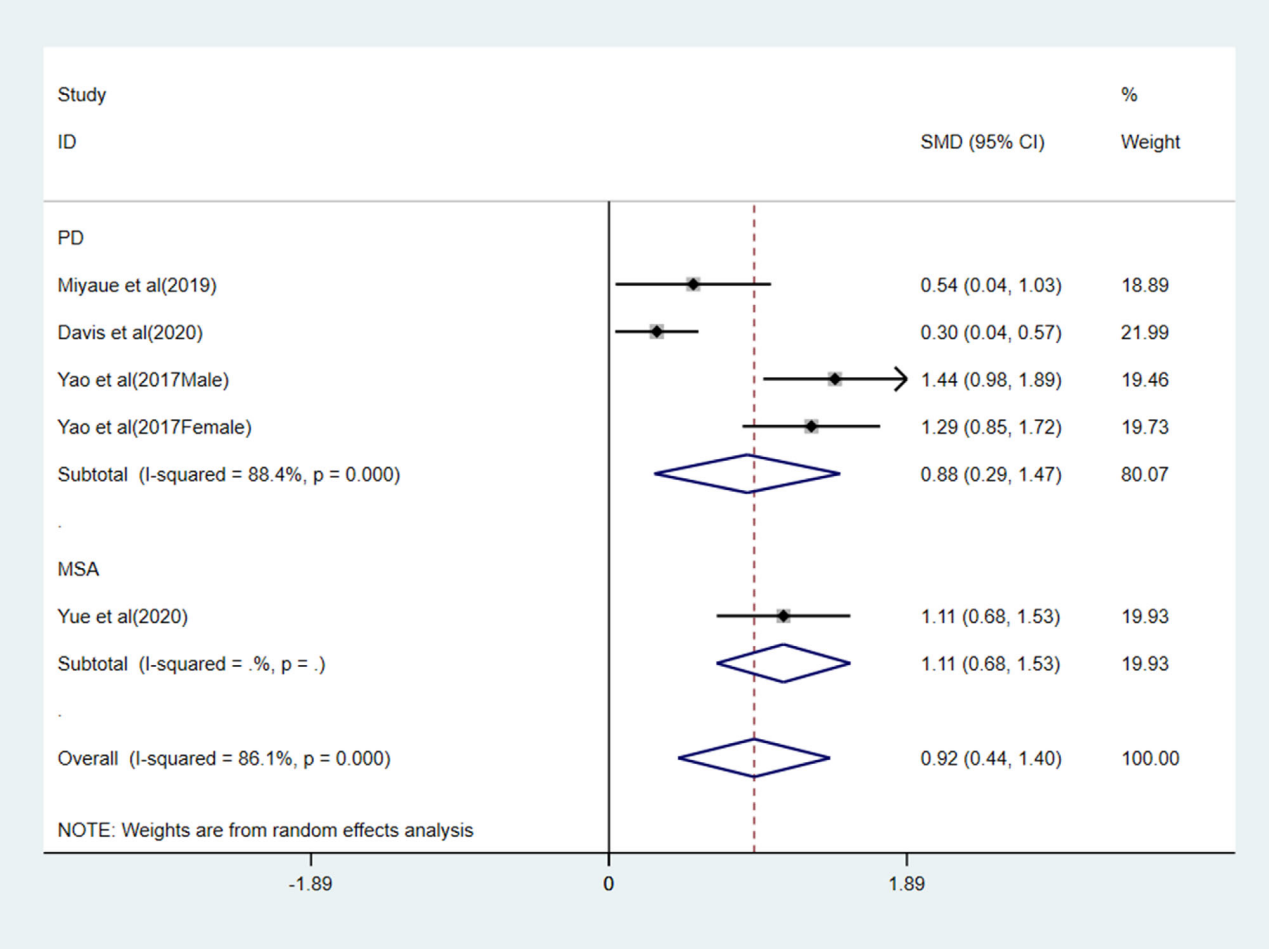
Forest plot of GDF15 levels with PD patients and MSA

**FIGURE 3:**
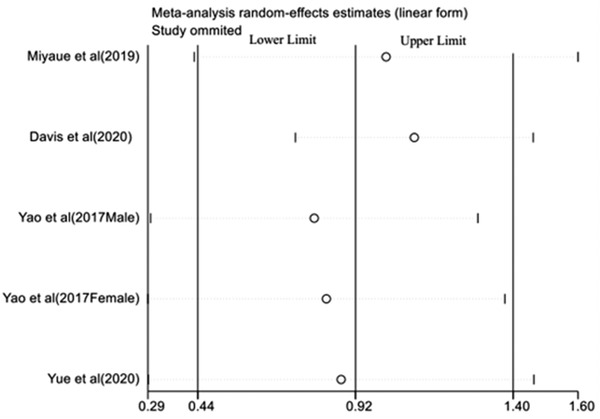
Sensitivity analysis of the pooled effect

Four studies reported the serum level of GDF15 in patients with neurodegenerative disease (three studies for PD, one studies for MSA). The pooled results of the random effect model indicated GDF15 levels were significantly higher in patients with neurodegenerative disease than healthy people (SMD = 0.92, 95% CI: 0.44–1.40, *Z *= 3.75, *p *< 0.001), with high heterogeneity among studies (*I* ^2 ^= 86.1%, *p *< 0.001). For patients with PD and MSA, the serum levels of GDF15 were all higher than healthy people (SMD = 0.88, 95% CI: 0.29–1.47, *Z *= 2.92, *p *=0 .03), (SMD = 1.11, 95% CI: 0.68–1.53, *Z *= 5.13, *p *< 0.001) (Figure 2). Sensitivity analysis results were shown in Figure 3; the pooled effect changed slightly by removing each study.

### 3.3 Sensitivity and specificity of GDF15

**FIGURE 4:**
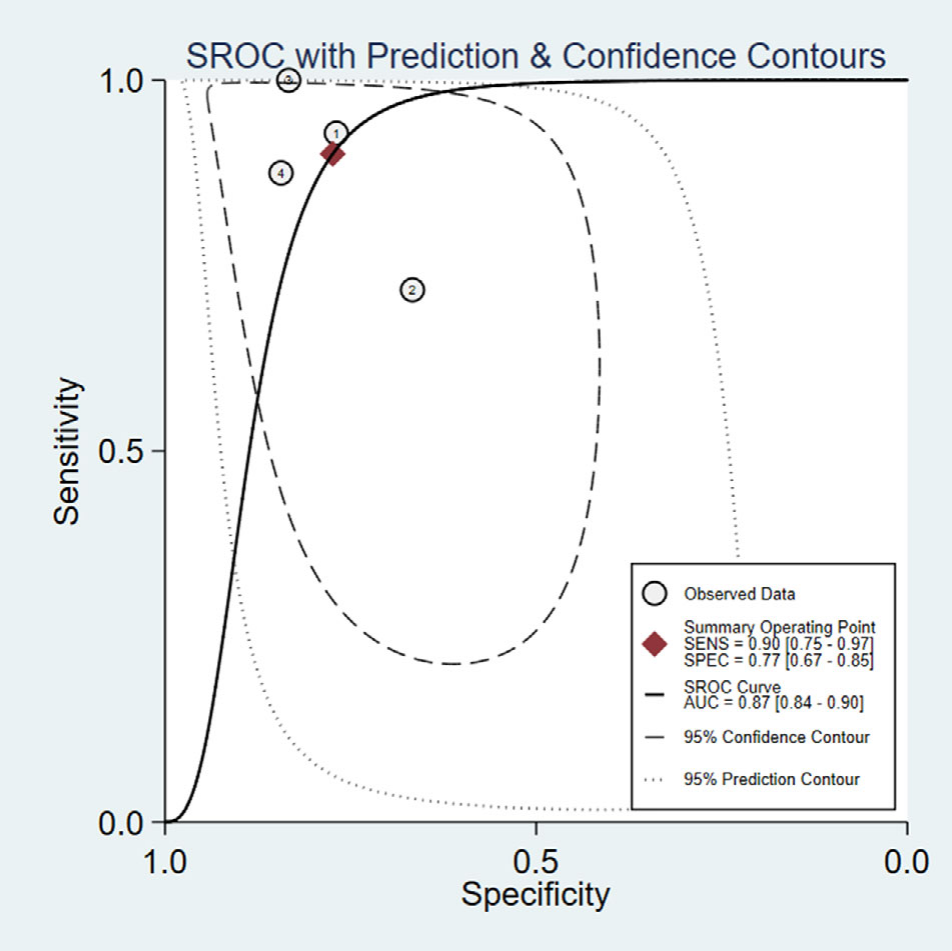
SROC curve of GDF15 for the diagnosis of neurodegenerative diseases

**FIGURE 5:**
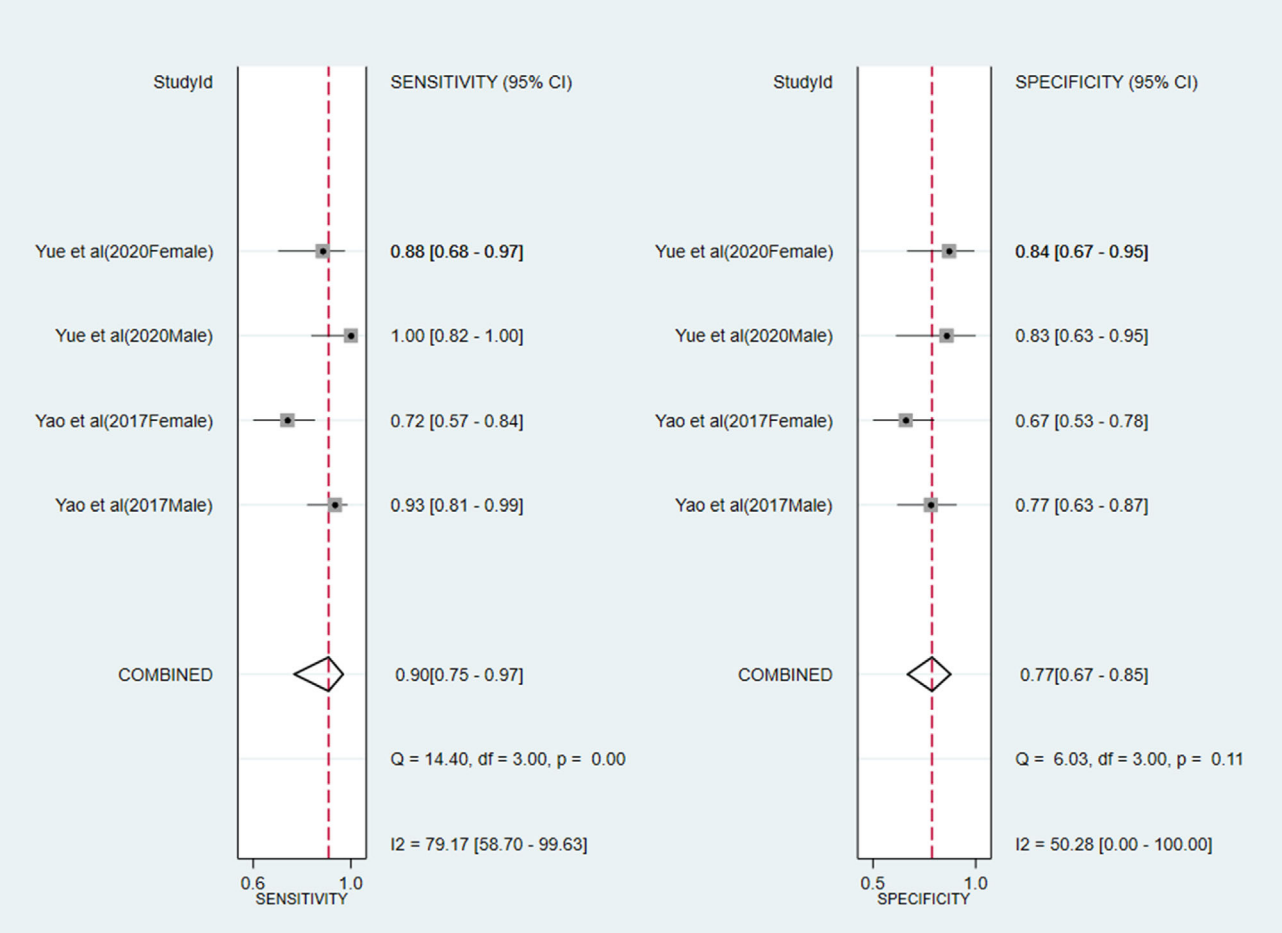
Forest plot of sensitivity and specificity biomarker of GDF15

Yue et al. (2020) suggested that serum GDF15 levels may be a potential diagnostic biomarker for multiple system atrophy patients compared with healthy controls and PD patients. Yao et al. (2017) have shown that GDF15 may be a potential biomarker for the diagnosis and monitoring of motor severity in PD. Two studies reported the diagnostic value of GDF15 for neurodegenerative disease in different genders. Compared with the healthy control, SROC for sensitivity and specificity indicated that the serum levels of GDF15 were a better marker for diagnostic utility of neurodegenerative disease. Sensitivity and specificity of biomarker of GDF15 were 0.90 (95% CI: 0.75–0.97), 0.77 (95% CI: 0.67–0.65) (Figure 4), and AUC = 0.87 (95% CI: 0.84–0.90) (Figure 5), respectively.

### 3.4 Systematic review

Four studies did not provide sufficient information for meta‐analysis. The study by Maetzler et al. (2016) indicated that CSF GDF15 had potential to distinguish Lewy body disorder patients from control. Also, the study indicated that adjusted GDF15 levels in PDD were significantly higher than that in PDND patients and intermediate in DLB patients. In patients with Lewy body disease, the levels of GDF15 were positively correlated with age at onset of PD and dementia, Hoehn & Yahr stage, and t‐Tau and p‐Tau levels in cerebrospinal fluid, and negatively correlated with Mini‐Mental State Examination. Fuchs et al. (2013) explored possible cross‐sectional and prospective correlations between serum levels of MIC1/GDF15 and cognitive impairment and decline. The results suggested a significant correlation between increasing levels of MIC1/GDF15 and cognitive impairment from normal to MCI or AD. The results also indicated that the serum levels of MIC1/GDF15 could be utilized as a potential marker to identify future cognitive impairment. A case‐control design by Chai et al. (2016) demonstrated that higher levels of GDF15 may be a biomarker for incognitive impairment no dementia (CICD) and AD in individual with white matter hyperintensities. However, Conte et al. (2020) suggested GDF15 levels were similar between AD patients and healthy control.

## 4 DISCUSSION

The purpose of this systematic review and meta‐analysis was to quantitatively assess the association between the GDF15 levels and neurodegenerative disease. Our study included neurodegenerative diseases such as AD, PD, and MSA. We found that GDF15 levels were higher in patient with neurodegenerative disease than that in the healthy people. For patients with PD and MSA, GDF15 levels were higher than that in healthy people. The diagnostic utility of GDF15 had a good sensitivity of 0.90 and had a good specificity of 0.77 in distinguishing neurodegenerative disease from health. These results demonstrated that GDF15 levels could be a potential marker for neurodegenerative disease. Several studies have reported that increased GDF15 levels were associated with PD, cognitive decline, AD, and Lewy body dementia (Chai et al., 2016; Kim et al., 2015; Yao et al., 2017). A review by Jiang et al. (2016) concluded that MIC‐1/GDF15 could be used as a marker of age‐related cognitive impairment and brain structural defects. These results were coincidence with our findings.

From a mechanistic view, GDF15 secretion response to mitochondrial stress was found in skeletal muscle. And mitochondrial dysfunction was observed in the skeletal muscle of patients with PD (Chung et al., 2017). Mitochondrial DNA defects and mutations caused mitochondrial dysfunction, which may play an important role in aging and neurodegeneration in PD (Bender et al., 2006; Kraytsberg et al., 2006). The amount of mitochondrial DNA deletions in the striatum of PD patients was significantly higher than that of healthy individuals (Ikebe et al., 1990). And mitochondrial dysfunction was associated with the neurodegenerative process of MSA (Fernagut et al., 2014; Jellinger, 2012). The variable combination of parkinsonism, cerebellar injury, and autonomic dysfunction were characteristic of MSA (Gilman et al., 2008). In addition to its role in mitochondrial dysfunction, GDF15 may play a role in inflammation. GDF15 was important for the neuroprotection of dopaminergic neurons in a mouse model of Parkinson's disease (Machado et al., 2016). If this protective effect applies to humans, the increased levels of GDF15 in PD patients may be considered as a reaction to inflammatory damage. Meanwhile, studies reported that GDF15 as part of the anti‐inflammatory cytokine network was upregulated in response to lesions and injury in the central nervous system (Breit et al., 2011; Johnen et al., 2012). In terms of cognition, the integrity of white matter was necessary for normal brain function containing cognition (Fields, 2008). Other studies have shown cognitive impairment in dementia and aging relation to white matter hyperintensities (Xu et al., 2015). And a study suggested that the increase of GDF15 in peripheral blood may be a marker of white matter hyperintensities‐related cognitive decline (Chai et al., 2016).

This meta‐analysis has some limitations. First, we only investigated the diagnostic validity using the SROC curve of GDF15 levels for total neurodegenerative disease. There were not sufficient studies for subgroup analysis of different disease types. Second, there was heterogeneity among the studies included in this meta‐analysis. This issue was balanced by utilizing the random‐effects model. However, a limited number of studies could not perform subgroup meta‐analysis for GDF15 levels in different parts and GDF15 levels in different diseases. Lastly, we cannot exclude the possibility of a publication bias (e.g., the tendency for studies to be more likely to be published if their results are positive than if they are negative or null).

In conclusion, this meta‐analysis found GDF15 as a potential marker of neurodegenerative disease. Compared with health control, GDF15 levels were significantly higher in PD, MSA, and AD. Higher‐quality studies on the relationship between the levels of GDF15 and neurodegenerative disease were needed aiming to better evaluate its clinical prognostic and diagnostic value.

## AUTHORSHIP

Hong Liu designed the literature search and analysis. Xin‐hong Xue and Lin‐Lin Tao searched the studies and performed the quality assessment of the results. Dao‐qing Su and Cun‐ju Guo analyzed the data and interpreted the result. Xin‐hong Xue wrote the manuscript. Xin‐hong Xue and Hong Liu finalized the manuscript. All authors discussed and reviewed and approved the final manuscript.

## CONFLICT OF INTEREST

The authors declare that they have no conflict of interests.

### PEER REVIEW

The peer review history for this article is available at https://publons.com/publon/10.1002/brb3.2502

## References

[brb32502-bib-0001] Abeliovich, A. , & Gitler, A. D. (2016). Defects in trafficking bridge Parkinson's disease pathology and genetics. Nature, 539(7628), 207–216. 10.1038/nature20414 27830778

[brb32502-bib-0002] Bender, A. , Krishnan, K. J. , Morris, C. M. , Taylor, G. A. , Reeve, A. K. , Perry, R. H. , Jaros, E. , Hersheson, J. S. , Betts, J. , Klopstock, T. , Taylor, R. W. , & Turnbull, D. M. (2006). High levels of mitochondrial DNA deletions in substantia nigra neurons in aging and Parkinson disease. Nature Genetics, 38(5), 515–517. 10.1038/ng1769 16604074

[brb32502-bib-0003] Breit, S. N. , Johnen, H. , Cook, A. D. , Tsai, V. W. W. , Mohammad, M. G. , Kuffner, T. , Zhang, H. P. , Marquis, C. P. , Jiang, L. , Lockwood, G. , Lee‐Ng, M. , Husaini, Y. , Wu, L. , Hamilton, J. A. , & Brown, D. A. (2011). The TGF‐β superfamily cytokine, MIC‐1/GDF15: A pleotrophic cytokine with roles in inflammation, cancer and metabolism. Growth Factors, 29(5), 187–195. 10.3109/08977194.2011.607137 21831009

[brb32502-bib-0004] Brown, D. A. , Ward, R. L. , Buckhaults, P. , Liu, T. , Romans, K. E. , Hawkins, N. J. , Bauskin, A. R. , Kinzler, K. W. , Vogelstein, B. , & Breit, S. N. (2003). MIC‐1 serum level and genotype: Associations with progress and prognosis of colorectal carcinoma. Clinical Cancer Research, 9(7), 2642–2650. 12855642

[brb32502-bib-0005] Canter, R. G. , Penney, J. , & Tsai, L.‐ H. (2016). The road to restoring neural circuits for the treatment of Alzheimer's disease. Nature, 539(7628), 187–196. 10.1038/nature20412 27830780

[brb32502-bib-0006] Carrillo‐García, C. , Prochnow, S. , Simeonova, I. K. , Strelau, J. , Hölzl‐Wenig, G. , Mandl, C. , Unsicker, K. , Von Bohlen Und Halbach, O. , & Ciccolini, F. (2014). Growth/differentiation factor 15 promotes EGFR signalling, and regulates proliferation and migration in the hippocampus of neonatal and young adult mice. Development (Cambridge, England), 141(4), 773–783. 10.1242/dev.096131 PMC3930467 24496615

[brb32502-bib-0007] Chai, Y. L. , Hilal, S. , Chong, J. P. C. , Ng, Y. X. , Liew, O. W. , Xu, X. , Ikram, M. K. , Venketasubramanian, N. , Richards, A. M. , Lai, M. K. P. , & Chen, C. P. (2016). Growth differentiation factor‐15 and white matter hyperintensities in cognitive impairment and dementia. Medicine, (33), e4566. 10.1097/md.0000000000004566 27537582 PMC5370808

[brb32502-bib-0008] Chung, H. K. , Ryu, D. , Kim, K. S. , Chang, J. Y. , Kim, Y. K. , Yi, H.‐S. , Kang, S. G. , Choi, M. J. , Lee, S. E. , Jung, S.‐B. , Ryu, M. J. , Kim, S. J. , Kweon, G. R. , Kim, H. , Hwang, J. H. , Lee, C.‐H.o , Lee, S.‐J. , Wall, C. E. , Downes, M. , … Shong, M. (2017). Growth differentiation factor 15 is a myomitokine governing systemic energy homeostasis. Journal of Cell Biology, 216(1), 149–165. 10.1083/jcb.201607110 PMC5223607 27986797

[brb32502-bib-0009] Conte, M. , Sabbatinelli, J. , Chiariello, A. , Martucci, M. , Santoro, A. , Monti, D. , Arcaro, M. , Galimberti, D. , Scarpini, E. , Bonfigli, A. R. , Giuliani, A. , Olivieri, F. , Franceschi, C. , & Salvioli, S. (2020). Disease‐specific plasma levels of mitokines FGF21, GDF15, and Humanin in type II diabetes and Alzheimer's disease in comparison with healthy aging. Geroscience, 43, 985–1001, 10.1007/s11357-020-00287-w 33131010 PMC8110619

[brb32502-bib-0010] Davis, R. L. , Wong, S. L. , Carling, P. J. , Payne, T. , Sue, C. M. , & Bandmann, O. (2020). Serum FGF‐21, GDF‐15, and blood mtDNA copy number are not biomarkers of Parkinson disease. Neurology Clinical Practice, 10(1), 40–46. 10.1212/cpj.0000000000000702 32190419 PMC7057070

[brb32502-bib-0011] Emmerson, P. J. , Duffin, K. L. , Chintharlapalli, S. , & Wu, X. (2018). GDF15 and growth control. Frontiers in Physiology, 9, 1712. 10.3389/fphys.2018.01712 30542297 PMC6277789

[brb32502-bib-0012] Fanciulli, A. , Stankovic, I. , Krismer, F. , Seppi, K. , Levin, J. , & Wenning, G. K. (2019). Multiple system atrophy. International Review of Neurobiology, 149, 137–192. 10.1016/bs.irn.2019.10.004 31779811

[brb32502-bib-0013] Fernagut, P.‐O. , Dehay, B. , Maillard, A. , Bezard, E. , Perez, P. , Traon, A. P.‐L. , Rascol, O. , Foubert‐Samier, A. , Tison, F. , & Meissner, W. G. (2014). Multiple system atrophy: A prototypical synucleinopathy for disease‐modifying therapeutic strategies. Neurobiology of Disease, 67, 133–139. 10.1016/j.nbd.2014.03.021 24727096

[brb32502-bib-0014] Fields, R. D. (2008). White matter in learning, cognition and psychiatric disorders. Trends in Neuroscience (Tins), 31(7), 361–370. 10.1016/j.tins.2008.04.001 PMC2486416 18538868

[brb32502-bib-0015] Fuchs, T. , Trollor, J. N. , Crawford, J. , Brown, D. A. , Baune, B. T. , Samaras, K. , Campbell, L. , Breit, S. N. , Brodaty, H. , Sachdev, P. , & Smith, E. (2013). Macrophage inhibitory cytokine‐1 is associated with cognitive impairment and predicts cognitive decline—The Sydney Memory and Aging Study. Aging Cell, 12(5), 882–889. 10.1111/acel.12116 23758647

[brb32502-bib-0016] Gilman, S. , Wenning, G. K. , Low, P. A. , Brooks, D. J. , Mathias, C. J. , Trojanowski, J. Q. , Wood, N. W. , Colosimo, C. , Durr, A. , Fowler, C. J. , Kaufmann, H. , Klockgether, T. , Lees, A. , Poewe, W. , Quinn, N. , Revesz, T. , Robertson, D. , Sandroni, P. , Seppi, K. , & Vidailhet, M. (2008). Second consensus statement on the diagnosis of multiple system atrophy. Neurology, 71(9), 670–676. 10.1212/01.wnl.0000324625.00404.15 18725592 PMC2676993

[brb32502-bib-0017] Hagström, E. , Held, C. , Stewart, R. A. H. , Aylward, P. E. , Budaj, A. , Cannon, C. P. , Koenig, W. , Krug‐Gourley, S. , Mohler, E. R. , Steg, P. G. , Tarka, E. , Östlund, O. , White, H. D. , Siegbahn, A. , & Wallentin, L. (2017). Growth differentiation factor 15 predicts all‐cause morbidity and mortality in stable coronary heart disease. Clinical Chemistry, 63(1), 325–333. 10.1373/clinchem.2016.260570 27811204

[brb32502-bib-0018] Heemels, M.‐T. (2016). Neurodegenerative diseases. Nature, 539(7628), 179. 10.1038/539179a 27830810

[brb32502-bib-0019] Ikebe, S.‐I. , Tanaka, M. , Ohno, K. , Sato, W. , Hattori, K. , Kondo, T. , Mizuno, Y. , & Ozawa, T. (1990). Increase of deleted mitochondrial DNA in the striatum in Parkinson's disease and senescence. Biochemical and Biophysical Research Communications, 170(3), 1044–1048. 10.1016/0006-291x(90)90497-b 2390073

[brb32502-bib-0020] Jellinger, K. A. (2012). Interaction between pathogenic proteins in neurodegenerative disorders. Journal of Cellular and Molecular Medicine, 16(6), 1166–1183. 10.1111/j.1582-4934.2011.01507.x 22176890 PMC3823071

[brb32502-bib-0021] Jiang, J. , Wen, W. , & Sachdev, P. S. (2016). Macrophage inhibitory cytokine‐1/growth differentiation factor 15 as a marker of cognitive ageing and dementia. Current Opinion in Psychiatry, 29(2), 181–186. 10.1097/yco.0000000000000225 26731555

[brb32502-bib-0022] Johnen, H. , Kuffner, T. , Brown, D. A. , Wu, B. J. , Stocker, R. , & Breit, S. N. (2012). Increased expression of the TGF‐b superfamily cytokine MIC‐1/GDF15 protects ApoE(‐/‐) mice from the development of atherosclerosis. Cardiovascular Pathology, 21(6), 499–505. 10.1016/j.carpath.2012.02.003 22386250

[brb32502-bib-0023] Kim, D. H. , Lee, D. , Chang, E. H. , Kim, J.i H. , Hwang, J. W. , Kim, J.u‐Y. , Kyung, J. W. , Kim, S. H. , Oh, J. S. , Shim, S. M. , Na, D. L. , Oh, W. , & Chang, J. W. (2015). GDF‐15 secreted from human umbilical cord blood mesenchymal stem cells delivered through the cerebrospinal fluid promotes hippocampal neurogenesis and synaptic activity in an Alzheimer's disease model. Stem Cells and Development, 24(20), 2378–2390. 10.1089/scd.2014.0487 26154268 PMC4598918

[brb32502-bib-0024] Kraytsberg, Y. , Kudryavtseva, E. , Mckee, A. C. , Geula, C. , Kowall, N. W. , & Khrapko, K. (2006). Mitochondrial DNA deletions are abundant and cause functional impairment in aged human substantia nigra neurons. Nature Genetics, 38(5), 518–520. 10.1038/ng1778 16604072

[brb32502-bib-0025] Machado, V. , Haas, S. J.‐P. , Von Bohlen Und Halbach, O. , Wree, A. , Krieglstein, K. , Unsicker, K. , & Spittau, B. (2016). Growth/differentiation factor‐15 deficiency compromises dopaminergic neuron survival and microglial response in the 6‐hydroxydopamine mouse model of Parkinson's disease. Neurobiology of Disease, 88, 1–15. 10.1016/j.nbd.2015.12.016 26733415

[brb32502-bib-0026] Maetzler, W. , Deleersnijder, W. , Hanssens, V. , Bernard, A. , Brockmann, K. , Marquetand, J. , Wurster, I. , Rattay, T. W. , Roncoroni, L. , Schaeffer, E. , Lerche, S. , Apel, A. , Deuschle, C. , & Berg, D. (2016). GDF15/MIC1 and MMP9 Cerebrospinal Fluid Levels in Parkinson's Disease and Lewy Body Dementia. Plos One, 11(3), e0149349. 10.1371/journal.pone.0149349 26938614 PMC4777571

[brb32502-bib-0027] Miyaue, N. , Yabe, H. , & Nagai, M. (2020). Serum growth differentiation factor 15, but not lactate, is elevated in patients with Parkinson's disease. Journal of the Neurological Sciences, 409, 116616. 10.1016/j.jns.2019.116616 31862518

[brb32502-bib-0028] Moher, D. , Shamseer, L. , Clarke, M. , Ghersi, D. , Liberati, A. , Petticrew, M. , Shekelle, P. , & Stewart, L. A. (2015). Preferred reporting items for systematic review and meta‐analysis protocols (PRISMA‐P) 2015 statement. Syst Rev, 4(1), 1. 10.1186/2046-4053-4-1 25554246 PMC4320440

[brb32502-bib-0029] Stang, A. (2010). Critical evaluation of the Newcastle‐Ottawa scale for the assessment of the quality of nonrandomized studies in meta‐analyses. European Journal of Epidemiology, 25(9), 603–605. 10.1007/s10654-010-9491-z 20652370

[brb32502-bib-0030] Strelau, J. , Strzelczyk, A. , Rusu, P. , Bendner, G. , Wiese, S. , Diella, F. , Altick, A. L. , Von Bartheld, C. S. , Klein, R. , Sendtner, M. , & Unsicker, K. (2009). Progressive postnatal motoneuron loss in mice lacking GDF‐15. Journal of Neuroscience, 29(43), 13640–13648. 10.1523/jneurosci.1133-09.2009 19864576 PMC3320210

[brb32502-bib-0031] Strelau, J. , Sullivan, A. , Böttner, M. , Lingor, P. , Falkenstein, E. , Suter‐Crazzolara, C. , Galter, D. , Jaszai, J. , Krieglstein, K. , & Unsicker, K. (2000). Growth/differentiation factor‐15/macrophage inhibitory cytokine‐1 is a novel trophic factor for midbrain dopaminergic neurons in vivo. Journal of Neuroscience, 20(23), 8597–8603. 10.1523/jneurosci.20-23-08597.2000 11102463 PMC6773071

[brb32502-bib-0032] Unsicker, K. , Spittau, B. , & Krieglstein, K. (2013). The multiple facets of the TGF‐β family cytokine growth/differentiation factor‐15/macrophage inhibitory cytokine‐1. Cytokine & Growth Factor Reviews, 24(4), 373–384. 10.1016/j.cytogfr.2013.05.003 23787157

[brb32502-bib-0033] Xu, X. , Hilal, S. , Collinson, S. L. , Chong, E. J. , Ikram, M. K. , Venketasubramanian, N. , & Chen, C. L. (2015). Association of magnetic resonance imaging markers of cerebrovascular disease burden and cognition. Stroke; A Journal of Cerebral Circulation, 46(10), 2808–2814. 10.1161/strokeaha.115.010700 26330446

[brb32502-bib-0034] Yao, X. , Wang, D. , Zhang, L. , Wang, L. , Zhao, Z. , Chen, S. , Wang, X. , Yue, T. , & Liu, Y. (2017). Serum growth differentiation factor 15 in Parkinson disease. Neurodegenerative Diseases, 17(6), 251–260. 10.1159/000477349 28787735

[brb32502-bib-0035] Yue, T. , Lu, H. , Yao, X. M. , Du, X. , Wang, L. L. , Guo, D. D. , & Liu, Y. M. (2020). Elevated serum growth differentiation factor 15 in multiple system atrophy patients: A case control study. World Journal of Clinical Cases, 8(12), 2473–2483. https://doi.org.10.12998/wjcc.v8.i12.2473 32607324 10.12998/wjcc.v8.i12.2473 PMC7322433

